# For Mammals, Loss of Yolk and Gain of Milk Went Hand in Hand

**DOI:** 10.1371/journal.pbio.0060077

**Published:** 2008-03-18

**Authors:** Richard Robinson

When it comes to nourishing their young, mammals are a motley crew. They all produce milk, but beyond that it gets interesting. The two groups of therian, or live-bearing, animals—eutherian (placental) and metatherian (marsupial) mammals—feed their young in utero, but differ in that marsupial pregnancies are extremely short. (And just to make things more confusing, marsupials have a placenta, but it’s more akin to a yolk sac than a true placenta.) On the other hand, the prototherians (monotremes)—which are largely extinct save for platypuses and echidnas—are about as different as they could be: they don’t develop placentas, and instead, like non-mammalian vertebrates such as birds, reptiles, and amphibians, lay eggs filled with yolk.

Non-mammalian vertebrates rely exclusively on nutritional resources derived primarily from nutrient-rich vitellogenin (VTG) proteins stored in egg yolk to sustain their offspring during embryonic development and early life. Mammals evolved alternative nutrient sources, such as placentation and lactation, to nurture their young. What molecular events allowed these radical differences in provisioning strategies to arise, and how could yolk-filled eggs have lost their central importance for nourishment of offspring? A new study by David Brawand, Walter Wahli, and Henrik Kaessmann suggests that in eutherians and metatherians, the emergence of milk-feeding liberated mammals from complete nutritional dependence on the egg, while the subsequent development of a placenta allowed for the complete loss of egg yolk nourishment.

In reptiles and birds, yolk is composed mainly of proteins, lipids, phosphorous, and calcium. Most of these essential nutrients are either transported by or contained within VTG, a complex protein made in the liver and packed into the egg. To determine how mammalian evolution may have affected vitellogenin production, the authors searched for vitellogenin genes (*VIT*s), functional or otherwise, in representative genomes from each branch of the mammalian family tree.

Using *VIT*gene sequences from chicken as the template, the authors found several corrupted remnants of *VIT* genes in three eutherians: humans, dogs, and armadillos. One such remnant bore the same inactivating mutation in all three species, suggesting it arose, and the gene became nonfunctional, before these three split, over 100 million years ago.

Turning to the metatherian branch, they found that three *VIT*s were present, but, as in eutherians, all had become useless over time through mutation. Comparison of sequences from the North American opossum and two species of Australian wallabies showed similar mutations in all, indicating they were inactivated before the split between the Australian and American marsupials about 70 million years ago. How long before? To find out, the authors performed a simulation of disabling mutation events. According to their results, one gene (*VIT3*) was lost about 170 million years ago, most likely after the prototherian/therian split, but before eutherians and metatherians diverged from each other. Another gene (*VIT1*) was lost about 30 million years after that, around the time of the eutherian/metatherian divergence, and the third (*VIT2*) was lost only 70–90 million years ago.

In the prototherian duck-billed platypus, two *VIT* sequences were found. *VIT1*was inactivated about 50 million years ago, but a second, corresponding to either *VIT2* or *VIT3*, appears to still be functional, since it contains no aberrant stop codons or frame-shifting mutations. In egg-bearing animals, the amount of VTG a creature makes is partly a function of how many gene copies it has, with more genes associated with more protein. Thus, the single gene of the monotremes likely explains why they continue to make yolk, while their mammalian cousins do not, but may also partly explain why their eggs are relatively yolk-poor, compared to birds and reptiles. True to their betwixt-and-between status, monotremes have no teats, and secrete their milk onto an abdominal patch for their young to feed on.

**Figure:**
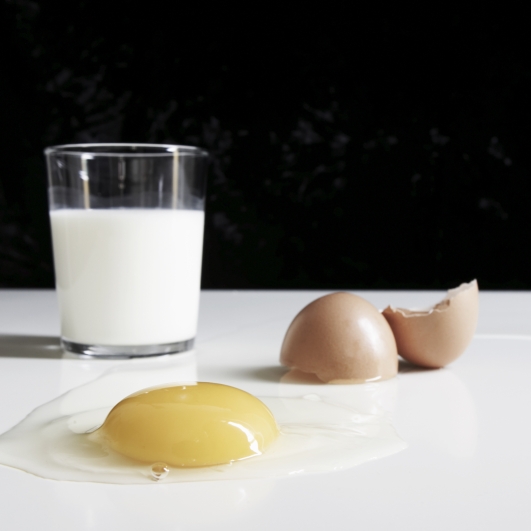
The emergence of alternative nourishment resources (lactation and placentation) during mammalian evolution set the stage for mammals' progressive loss of egg yolk nourishment (as a consequence of the loss of egg yolk genes). (Photo: Rasmus Kaessmann. http://www.kaessmannphotography.com/)

But nutrients lost from the egg must be provided elsewhere, and it was the development of lactation, the authors propose, that allowed the yolk-making *VIT* genes to go by the wayside. In their screen of the platypus genome, they identified three putative genes for casein, the milk protein. Casein binds calcium, an essential nutrient for growing young, whether in the egg or suckling after birth, and a nutrient that would be scarce in the absence of VTG. Based on the position of the genes and several features of their sequence, the authors propose these genes arose in the common ancestor of all mammals, before the three branches diverged. The emergence of casein, they suggest, “might thus have contributed to the increasing role for lactation for the nourishment of the young.”

Lactation, which arose simply to keep eggs moist, thus became an important alternative food source. Since the egg would no longer be the sole source of nutrition, the selective pressure on the VTG-expressing genes to remain functional would ease. Later, the development of the placenta and more advanced lactation physiology (in therians) allowed total loss of the *VIT* genes, completing the transition away from dependency on yolk. But what propelled mammals along their unique evolutionary path, this study shows, is the one feature shared by all members of this motley crew—they got milk.

